# Change of Direction Ability Performance in Cerebral Palsy Football Players According to Functional Profiles

**DOI:** 10.3389/fphys.2015.00409

**Published:** 2016-01-06

**Authors:** Raúl Reina, Jose M. Sarabia, Javier Yanci, María P. García-Vaquero, María Campayo-Piernas

**Affiliations:** ^1^Sports Research Centre, Miguel Hernández UniversityElche, Spain; ^2^Faculty of Physical Activity and Sports Science, University of the Basque Country, UPV/EHUVitoria-Gasteiz, Spain

**Keywords:** agility, classification, impairment, performance, paralympics

## Abstract

The aims of the present study were to evaluate the validity and reliability of the two different change of direction ability (CODA) tests in elite football players with cerebral palsy (CP) and to analyse the differences in performance of this ability between current functional classes (FT) and controls. The sample consisted of 96 international cerebral palsy football players (FPCP) and 37 football players. Participants were divided into four different groups according to the International Federation of Cerebral Palsy Football (IFCPF) classes and a control group (CG): FT5 (*n* = 8); FT6 (*n* = 12); FT7 (*n* = 62); FT8 (*n* = 14); and CG (*n* = 37). The reproducibility of Modified Agility Test (MAT) and Illinois Agility Test (IAT) (ICC = 0.82–0.95, SEM = 2.5–5.8%) showed excellent to good values. In two CODA tests, CG performed faster scores compared with FPCP classes (*p* < 0.01, *d* = 1.76–3.26). In IAT, FT8 class comparisons regarding the other classes were: FT5 (*p* = 0.047, *d* = 1.05), FT6 (*p* = 0.055, *d* = 1.19), and FT7 (*p* = 0.396, *d* = 0.56). With regard to MAT, FT8 class was also compared with FT5 (*p* = 0.006, *d* = 1.30), FT6 (*p* = 0.061, *d* = 0.93), and FT7 (*p* = 0.033, *d* = 1.01). No significant differences have been found between FT5, FT6, and FT7 classes. According to these results, IAT and MAT could be useful and reliable and valid tests to analyse CODA in FPCP. Each test (IAT and MAT) could be applied considering the cut point that classifiers need to make a decision about the FT8 class and the other FT classes (FT5, FT6, and FT7).

## Introduction

Football for people with cerebral palsy (CP) is a 7-a-side game with two 30 min halves (Kloyiam et al., 2011; Cámara et al., 2013). This is one of the 23 sports included in the programme of the next Paralympic Games in Rio 2016. The Fédération Internationale de Football Association (FIFA) laws of the game apply with some exceptions made by the new (1 January 2015) International Federation of Cerebral Palsy Football (IFCPF). Some of the changes include a smaller pitch and goal posts, no offside rule and players rolling the ball into play instead of a throw in (IFCPF, 2015a). This sport became independent on, under the umbrella of the IFCPF. On the field, teams are made up of seven ambulant CP players.

All players participating in official events must have an IFCPF classification. IFCPF has a classification system which allows all ambulant athletes with CP and related neurological conditions to take part. Paralympic classification systems aim to promote participation in sport by people with disabilities by controlling for the impact of impairment on the outcome of competition (Tweedy et al., 2014). The language and structure of the International Classification of Functioning, Disability, and Health (World Health Organization, 2012) is central to Paralympic classification, and the concepts of impairment and activity limitation are particularly important (Tweedy and Vanlandewijck, 2011). The International Paralympic Committee (IPC) recognizes eight eligible physical impairments in Paralympic sport: five impairments of function (i.e., impaired strength, impaired range of movement, hypertonia, ataxia, and athetosis) and three impairments of structure (i.e., limb deficiency, leg length difference, and short stature). In addition, the activities of focus are the Paralympic sports in which athletes compete. It is not mandatory for Paralympic sports to provide classification systems that cater to all eight physical impairment types (Tweedy et al., 2014). For example, CP Football is for athletes with hypertonia, ataxia, or athetosis of cerebral origin (e.g., cerebral palsy, traumatic brain injury, or stroke). Although this sport is governed by an international federation which includes cerebral palsy in its definition, the classification unit in Paralympic sport is the impairment, and it is related to several health conditions, not only cerebral palsy.

Based on these eligible impairments, CP Football has four classes based on the traditional Cerebral Palsy International Sports and Recreation Association (CPISRA) classification system, that is, four classes (C1–C4) for wheelchair athletes and the other four (C5–C8) for ambulant athletes (Reina, 2014). Applied to CP-football, the last four classes (FT) appear in the rules as: “(a) Class FT5: Diplegia, asymmetric diplegia, double hemiplegic, or dystonic. It includes moderate involvement with spasticity grade 2–3; involvement of both legs which may require orthotics/splints for walking; an asymmetric diplegia or double hemiplegic athlete with involvement on both sides with the lower limbs more affected than the upper extremities; or athletes with dystonia where the lower limbs are more affected than the upper extremities. (b) Class FT6: Athetosis, dystonic, ataxic or mixed cerebral palsy or related neurological conditions. It includes moderate involvement in all four limbs; the athlete ambulates without assistive devices but might require orthotics/splints; athetosis, dystonia, or ataxia is typically the most prevalent factor but some athletes can have problems with athetosis or ataxia mixed with spasticity; athletes with dystonic athetosis in all four limbs belong in this classification unless the impairment is minimal. (c) Class FT7. Hemiplegic, including spasticity grade 2–3 in one half of the body (on the frontal plane); athletes walk/run with a clearly noticeable limp due to spasticity in the lower limb; hemi gait pattern 2, 3, or 4 as per grouping described in gait patterns in spastic hemiplegia in children and young adults. They usually have a good functional ability on the other side of the body. (d) Class FT8. Diplegia, asymmetric diplegia, double hemiplegia, and/or dystonia. It includes hemiplegia with spasticity grade 1–2; monoplegia with spasticity grade 1 or 2 in a major joint in the lower limbs; athetosis, dystonia, ataxia or mixed cerebral palsy or other neurological conditions.”

In Paralympic sport, an evidence-based system of classification is one in which the system has a clearly stated purpose, and methods used for assigning class will achieve the stated purpose (Tweedy and Vanlandewijck, 2011). Although evidence-based methods for classifying impairments must primarily use valid and reliable measures of impairment, such measures cannot be the sole basis of classification (Beckman and Tweedy, 2009). This is because, although eligible impairments are permanent, many types of impairment are, to varying degrees, responsive to training. In most circumstances, current best practice requires classification panels to assign a class by collectively considering outcomes from the impairment assessment, together with three other forms of assessment [International Paralympic Committee (IPC), 2015]:(a) novel motor tasks, which are tasks that are unlikely to have been practiced by the athlete in the usual course of training for his or her sport; (b) sport-specific activities that are likely to have been frequently practiced by athletes training for the sport; and (c) a detailed training history and other personal and environmental factors likely to affect sports proficiency.

Football players are required to turn, sprint and change pace during matches (Stølen et al., 2005). Indeed, frequent variation in activities has been reported during a competitive match in elite football players (Bloomfield et al., 2007). During a football game, ~1300 changes in activity are undertaken in off-the-ball conditions (Stølen et al., 2005). Therefore, due to the relevance of change of direction ability (CODA) in this sport, the examination of the nature of this activity and its evaluation is one of the objectives of this study. Evaluating CODA in football has entailed the use of many different tests including *T*-test (Sporis et al., 2010; Chaouachi et al., 2012) *T*-test modifications (Sassi et al., 2009) and Illinois test (Miller et al., 2006). However, despite the fact that CODA has been evaluated in football players (Sporis et al., 2010; Chaouachi et al., 2012) children and adolescents with CP (Verschuren et al., 2009, 2010) and can be a determining performance component in CP Football, to our knowledge, no scientific articles have been published to determine the CODA in elite football players eligible for CP football, and its relationships with current classification profiles.

The aims of the present study were, firstly, to evaluate the reliability of the CODA measured by modified agility test (MAT) and Illinois agility test (IAT) in international football players with cerebral palsy (FPCP); secondly, to evaluate the validity of both CODA tests to check activity limitation of cerebral palsy football players (FPCP) regarding controls; and thirdly, to analyse the differences in this ability between different current IFCPF functional classes. Due to the different CP motor alterations (Unnithan et al., 1998) and that different types of tests used may determine the performance (Chaouachi et al., 2012) our working hypothesis is that there may be differences in the CODA between CPISRA functional classes, and also between FPCP and football players.

## Materials and methods

### Participants

Ninety-nine international FPCP and 37 football players took part in this study (*n* = 136). Written informed consent was obtained from the participants and their coaches, and data collection was conducted during 2013 CPISRA Intercontinental Cup (ICUP) qualifying tournament for the World Championships in 2015, while the control group was measured 1 month before the end of the regular league. Sixteen teams took part in the CPISRA ICUP, and players from 10 national teams voluntarily participated in the data collection. Reported competition experience indicated that 24.7% of players from FPCP group participated in the last Paralympics Games (London 2012). Football players had national level competition, and this control group (CG) was built up considering the mean age and training activity (Table 1). The study was approved by the institutional review committee (DPS.RRV.01.14) of the Miguel Hernández University (Elche, Spain) and conformed to the recommendations of the Declaration of Helsinki.

**Table 1:** **Descriptive data of elite football players with cerebral palsy (FPCP) and football players (CG)**.

**Class**	***n***	**Age (yr)**	**Mass (kg)**	**Height (cm)**	**BMI (kg·*m*^−2^)**	**Sport experience**	**Football sessions**	**Gym sessions**
FT5	8	23.2 ± 6.4	67.0 ± 7.5	175.9 ± 6.1	21.7 ± 2.7	11.4 ± 5.2	4.6 ± 1.2	3.0 ± 1.3
FT6	12	27.1 ± 8.9	65.6 ± 6.5	175.3 ± 4.7	21.5 ± 1.5	11.0 ± 3.6	2.6 ± 1.2	2.7 ± 1.1
FT7	64	24.8 ± 6.2	68.3 ± 8.5	175.1 ± 7.6	22.5 ± 2.9	9.8 ± 6.9	3.2 ± 1.3	2.7 ± 1.5
FT8	15	26.5 ± 7.6	73.2 ± 7.9	176.7 ± 8.9	23.5 ± 2.2	13.6 ± 9.6	3.1 ± 0.9	3.5 ± 1.7
CG	37	19.6 ± 3.4	72.6 ± 7.8	178.0 ± 5.9	22.9 ± 1.7	10.2 ± 5.1	3.4 ± 0.5	1.7 ± 0.9
Sample	136	23.7 ± 6.6	69.8 ± 8.2	176.0 ± 7.1	22.6 ± 2.5	10.5 ± 6.5	3.3 ± 1.1	2.5 ± 1.5

*BMI, Body Mass Index; Sport experience expressed in years; Football and Gym (strength) sessions expressed by times/week*.

### Procedures

A standardized warm-up was performed, consisting of a 5 min self-paced low-intensity run, skipping exercises, strides and two 15 m sprints with and without changes of direction. Information about the protocols was sent in advance to the teams, and participants practiced test protocols twice before data collection. Each participant performed anthropometric measures and two trials of two agility tests: IAT and MAT. A 3 min rest period was given between each trial (Mayhew et al., 1995). The best score in each test was used for data analysis.

#### Anthropometric measurements

The players' heights were measured using a stadiometer with an accuracy of ±1 mm (Harpenden, Holtain® Ltd., Crosswell, UK). Electronic scales (Oregon Scientific®, GR101, Portland, USA) with an accuracy of ± 0.01 kg were used to measure the body mass.

#### Illinois agility test

**Figure 1:**
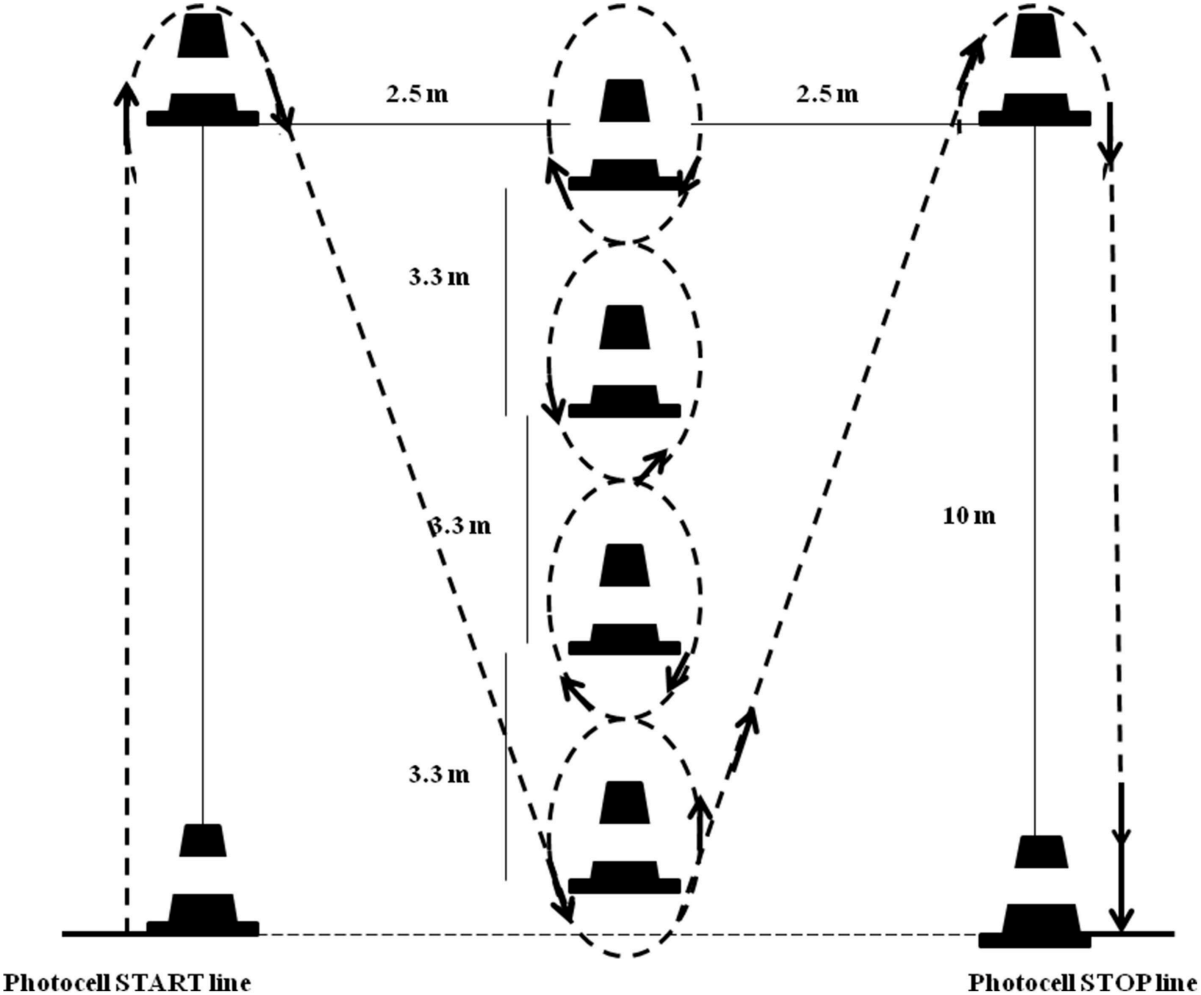
**Schematic representations for Illinois Agility Test (IAT)**.

The IAT is set up with four cones forming the agility area (Figure 1). On command, (1) the athlete sprints 10 m, turns and (2) returns to the starting line. After returning to the starting line, (3) he swerves in and out of four cones (4, 5) completing two 10 m sprints to finish the agility course (Miller et al., 2006). Performances were recorded using an electronic timing system (Globus®, Codogne, Italy). The infrared timing gates were positioned at the start and the finish lines at a height of 1 m. No technical advice was given as to the most effective movement technique. Subjects were only instructed to complete the test as quickly as possible. Subjects were instructed not to jump over the markers; they were to run around them, and the trial was not valid if they touched or toppled a marker.

#### Modified agility test

**Figure 2:**
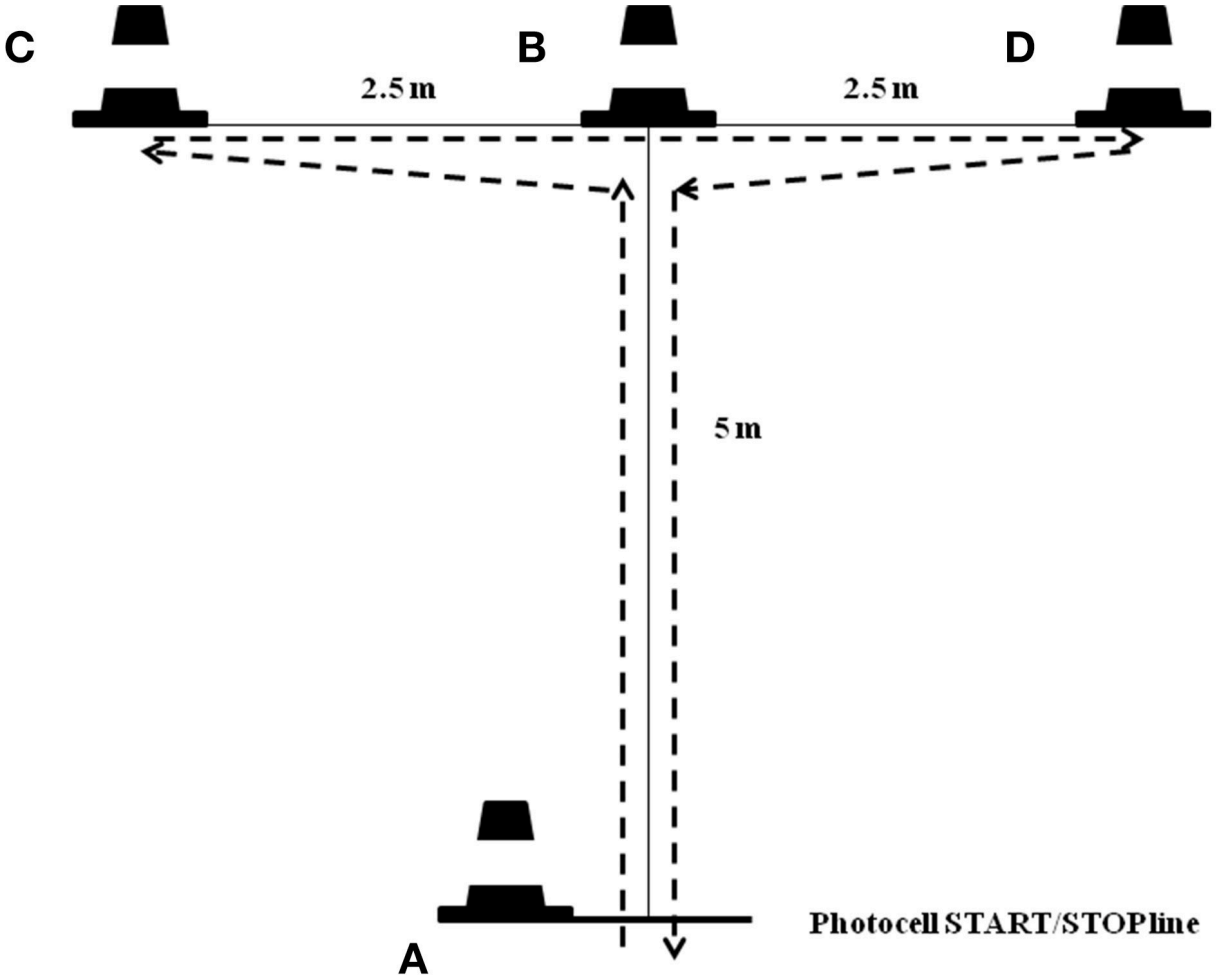
**Schematic representations for Modified Agility Test (MAT)**.

This test was originally described as a measure of four-directional agility and body control that evaluates the ability to change directions rapidly while maintaining balance without loss of speed (Semenick, 1990). The MAT was proposed by Sassi et al. (2009) and Pauole et al. (2000), and recently adapted by Yanci et al. (2013). This is considered a short duration test where linear movement in the antero-posterior and medio-lateral directions is required (Sassi et al., 2009). A previous study conducted with football players showed excellent MAT test reproducibility values (CV = 2.3%) (Yanci et al., 2014). The participants' movements during the MAT were as follows (Figure 2): (i) A–B movement (5 m): participants sprinted forward to cone B and touched the top of it with the right hand; (ii) B–C movement (2.5 m): moving laterally without crossing the feet, participants ran to cone C and touched the top of it with the left hand; (iii) C–D movement (5 m): participants ran laterally to cone D and touched the top of it with the right hand; (iv) D–B movement (2.5 m): participants moved back to cone B and touched the top of it with the left hand; (v) B–A movement (5 m): participants ran backward to line A. Start position was standardized, with the preferred foot close to the start line. The test was repeated if the athlete crossed one foot in front of the other, did not touch the cone and failed to face forward throughout. MAT performances test were recorded using an electronic timing system (Globus®, Codogne, Italy) positioned at the start line at a height of 1 m.

### Data analysis

Results are presented as mean ± standard deviation (SD), and coefficient of variation (CV) was also calculated using next formula: *CV* = (*SD*/*Mean* · 100) (Atkinson and Nevill, 1998). Reliability among two trials in each agility test was assessed using intra-class correlations (ICC) and Standard Error Measurement (SEM). ICC > 0.90 were considered excellent, 0.75–0.90 good and < 0.75 as poor to moderate (Portney and Watkins, 2008). The SEM was calculated by using the following formula: $SEM=SD\cdot \sqrt{1-ICC}$. Strength of association between two agility tests used in this study was assessed using a Pearson correlation (r). To interpret those results the threshold values for Pearson product-moment used by Salaj and Markovic (2011) were used: low (*r* ≤ 0.3), moderate (0.3 < *r* ≤ 0.7) to high (*r* > 0.7). A spreadsheet designed by Hopkins (2015) was used to evaluate changes in scores (bias) between testing repetitions.

Kolmogorov test was applied to evaluate the normal data distribution. All analyzed variables had a normal distribution, and parametric statistic was used. Atypical scores were evaluated for those players scoring above 95% interval of confidence, and one player from the class FT6 and two players from the class FT7 have been removed from the data analysis. A One-way analysis of variance (ANOVA) with least significant difference *post-hoc* comparison (Scheffé correction) was used to examine the mean differences between groups and FPCP sub-groups. Cohen's effect sizes (*d*) between groups were also calculated (Cohen, 1988). Interpretation of effect sizes for highly trained athletes was: above 1.0, between 0.5 and 1.0, between 0.25 and 0.5 and lower than 0.25 were considered as large, moderate, small and trivial, respectively (Rhea, 2004). Data analysis was performed using the Statistical Package for Social Sciences (version 21.0 for Windows, SPSS Inc, Chicago, IL, USA). Statistical significance was set at an alpha level of *p* < 0.05.

## Results

Due to the different classification profiles about the players who took part in the study, a within-group correlation analysis was conducted between two CODA tests. Within-session reliability for each player was evaluated among two trials performed. IAT reliability was ICC = 0.96 (0.91, 0.98) and SEM = 2.5% (2.23, 2.86) for the FPCP group, and ICC = 0.84 (0.73, 0.91) and SEM = 1.88% (1.57, 2.38) for CG. CV for CG was 4.21%, and higher in the FPCP classes: FT8 = 7.01%, FT7 = 9.48%, FT6 = 6.85%, and FT5 = 10.37%. The change in mean scores (bias) were −0.14 s (−0.26, −0.02) for FPCP group and 0.01 s (−0.12, −0.14) for CG respectively. On the other hand, FPCP group showed a reliability in MAT of ICC = 0.82 (0.75, 0.87) and SEM = 5.84% (5.20, 6.68), while CG was ICC = 0.76 (0.61, 0.86) and SEM = 3.02% (2.53, 3.78). Similar results were obtained regarding CV: CG = 6%, FT8 = 10.01%, FT7 = 11.43%, FT6 = 17.09%, and FT5 = 16.81%. The bias for this test was −0.25 s (−0.37, −0.12) for FPCP group and −0.17 s (−0.24, −0.09) for CG respectively.

Overall correlation analysis between two CODA tests showed a significant and positive relationship (*r* = 0.736, *p* < 0.001); both FPCP (*r* = 0.555, *p* < 0.001) and CG (*r* = 0.453, *p* < 0.01). However, if we analyse this relationship between the different classes in the FPCP group, correlation is not significant for classes FT5 (*r* = 0.359, *p* = 0.383), while it is significant for classes FT6 (*r* = 0.838, *p* < 0.01), FT7 (*r* = 0.517, *p* < 0.001), and FT8 (*r* = 0.641, *p* < 0.01).

**Figure 3:**
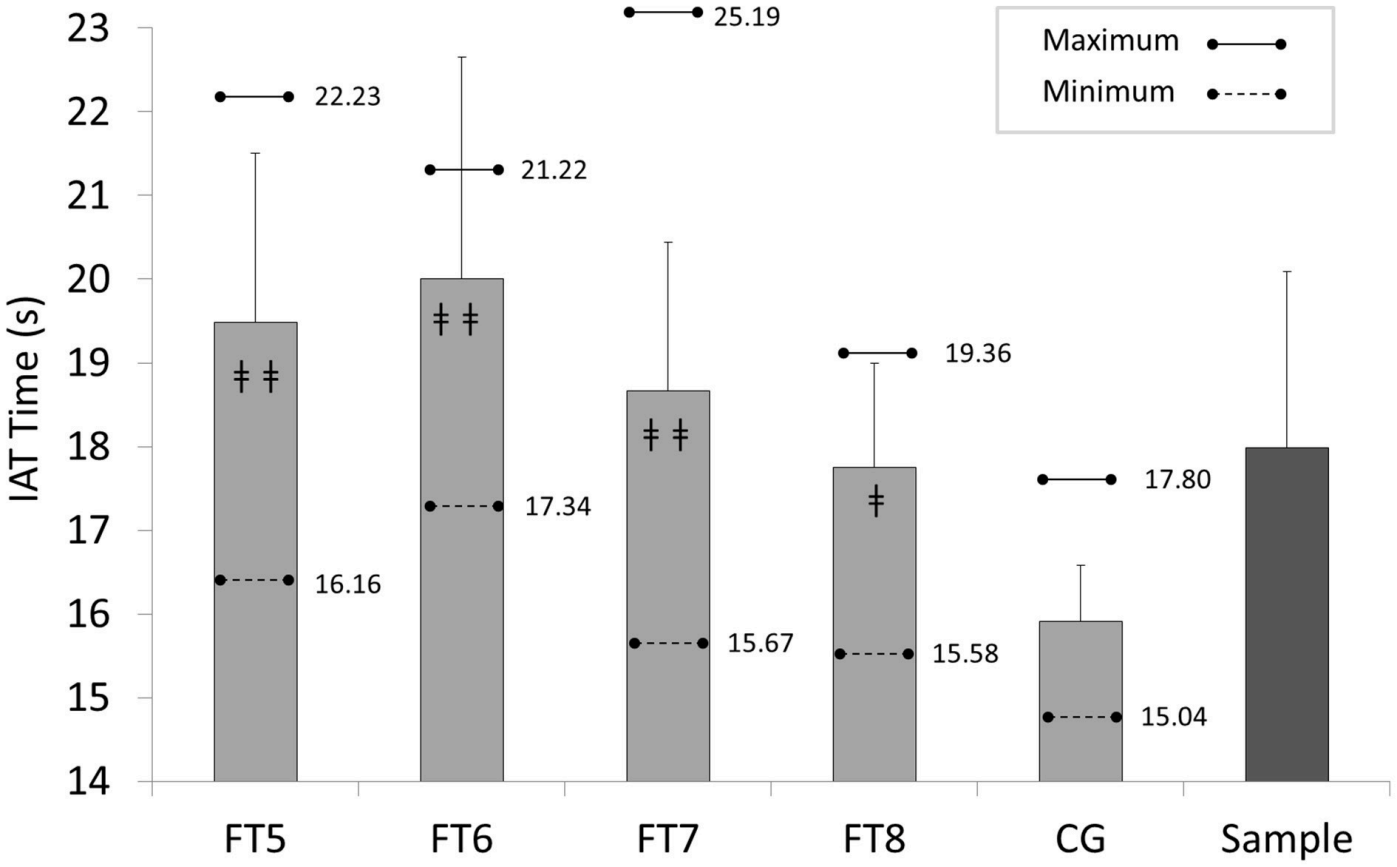
**Descriptive scores for each group in Illinois Agility test (IAT): FT5, FT6, FT7, and FT8, Football Players with Cerebral Palsy Classes; CG, control group; ‡‡, CG vs. FT5, FT6, or FT7, *p* < 0.001; ‡, CG vs. FT8, *p* < 0.01**.

**Figure 4:**
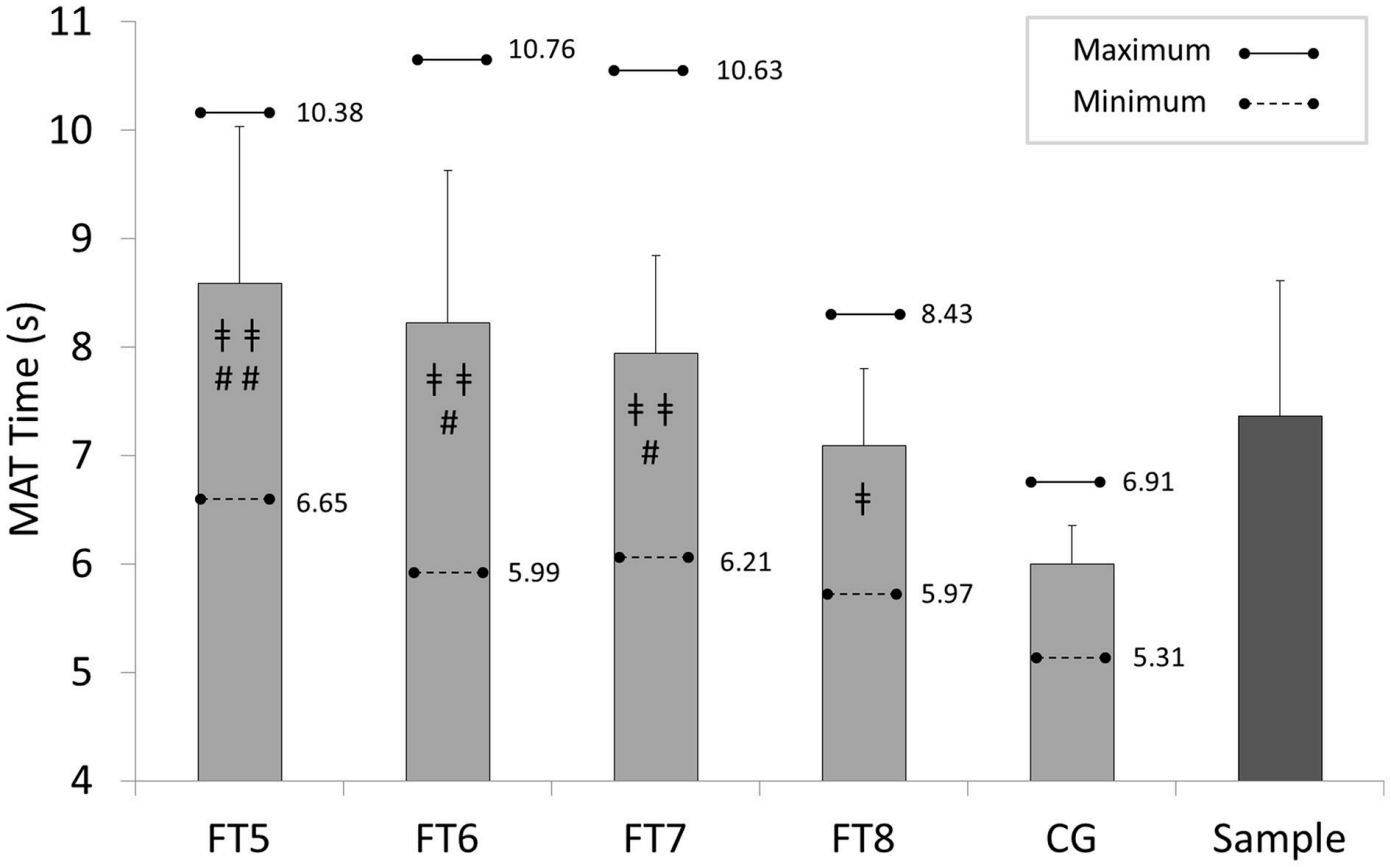
**Descriptive scores for each group in Modified Agility test (MAT); FT5, FT6, FT7, and FT8, Football Players with Cerebral Palsy Classes; CG, control group; ‡‡, CG vs. FT5, FT6, or FT7, *p* < 0.001; ‡, CG vs. FT8, *p* < 0.01; ##, FT8 vs. FT5, *p* < 0.01; #, FT8 vs. FT6-FT7, *p* < 0.05**.

Mean, standard deviations, maximum and minimum scores for each group and overall are reported in Figures 3, 4. In IAT, CG performed faster scores (15.91 ± 0.67 s), compared with FT8 (17.75 ± 1.24 s; *d* = 1.84, large), FT7 (18.67 ± 1.77 s; *d* = 2.06, large), FT6 (19.34 ± 1.32 s; *d* = 3.27, large), and FT5 (19.49 ± 2.02 s; *d* = 2.38, large) respectively. Similar results were obtained for MAT: CG (5.99 ± 0.36 s), FT8 (7.09 ± 0.71 s; *d* = 1.95, large), FT7 (7.94 ± 0.91 s; *d* = 2.82, large), FT6 (8.22 ± 1.41 s; *d* = 2.17, large), and FT5 (8.59 ± 1.44 s; *d* = 2.48, large).

One-way ANOVA showed significant differences between groups, both IAT [*F*_(4, 128)_ = 36.93; *p* < 0.001] and MAT [*F*_(4, 129)_ = 26.30; *p* < 0.001]. Pair comparisons also report significant differences between CG and all FPCP classes (*p* < 0.001 regarding FT7, FT6, and FT5; *p* < 0.01 regarding FT8; *d* > 1.0, large).

In IAT, FT8 class comparisons regarding the other classes were: FT5 (*p* = 0.128; *d* = 1.03, large), FT6 (*p* = 0.124; *d* = 1.23, large), and FT7 (*p* = 0.325; *d* = 0.60, moderate). With regard to MAT, FT8 players showed significant differences regarding all the other FPCP groups: FT5 (*p* = 0.005; *d* = 1.32, large), FT6 (*p* = 0.034; *d* = 1.01, large), and FT7 (*p* = 0.026; *d* = 1.04, large). No significant differences have been found between FPCP classes FT5, FT6 and FT7.

## Discussion

The aim of this study was to compare the performance of FPCP and football players in two CODA tests, and analyse its relationship with current functional classification profiles in CP-football. To our knowledge, there is no study in the literature of the CODA in football players with CP and other related neurological conditions eligible for CP-football. According to IPC's Classification Policy, the development of evidence-based classification procedures is necessary, and tis check the activity limitation in sporting skills due to eligible impairment.

The analysis of the reproducibility of MAT and IAT showed excellent to good values, as other studies which used T-design CODA tests (Pauole et al., 2000; Sassi et al., 2009; Yanci et al., 2014) and IAT (Miller et al., 2006; Lockie et al., 2013). Also, high to moderate correlations have been obtained between both tests. However, FPCP groups showed CVs in MAT higher than 10% (FT8 = 10.01%, FT7 = 11.43%, FT6 = 17.09%, and FT5 = 16.81%). Future studies could include three trials of the test to improve CV, although SEM and ICC values were good. On the other hand, this test could be considered a “novel” task for the players (International Paralympic Committee (IPC), 2015).

A test that assesses linear acceleration, in addition to the ability to make several sharp cuts while continuing to sprint forward over specific distances, has value for field sports (Lockie et al., 2013). IAT involves acceleration, as well as directional changes when sprinting in a linear fashion. In the same way, CODA is considered a determinant for successful performance in football (Chaouachi et al., 2012, 2014) and the response to different, short and rapid movements is essential in football players. For this reason, CODA has been tested in order to assess football players' conditioning (Chaouachi et al., 2012; Yanci et al., 2014; Reilly et al., 2000). In spite of the fact difficulty in turning and stopping are characteristics of IFCPF (2015a) to the best of our knowledge, this is the first study of such ability in international FPCP. Both IAT and MAT tests showed that best score in CG is better than the best score in FPCP classes, and both tests could be valid and reliable to evaluate CODA activity limitation due to eligible impairments.

Current CP Football rules state that at least one player from the classes FT5 or FT6 should be on the field of play during the game, and teams cannot play with more than one player of class FT8. This rule is expected to change after the Rio Paralympic Games, and the IFCPF Board have suggested increasing to two FT5 or FT6 players in the lineup. This rule change will have an impact on team management and training, because these two classes are colloquially named “lower classes.” Then, the general performance of a team could be influenced by the classification of its players. In other words, a player with “moderate” or “mild” spastic diplegia could be classified as FT5 or FT8, with a major impact on team play or team squad (Reina, 2014). According to Tweedy et al. (2014), to assess the relative strength of association between valid measures of impairment and measures of performance, development of valid measures of impairment must be complemented by the development of standardized, sport-specific measures of performance, and tests applied in this study could be helpful in CP Football classification.

In our study, FT8 (“mild impairment” regarding the other three classes) showed significant differences with FT5, FT6, and FT7 in IAT (large effect sizes); but not in MAT, but with large (FT5 and FT6) and moderate (FT7) effect sizes. Players from class FT5 (e.g., spastic diplegia) presented the worst scores in both tests. Spastic diplegia manifests as high and constant “tightness” or “stiffness” in the muscles of the lower extremities, usually on the legs, hips, and pelvis. The abnormally high muscle tone that results creates lifelong difficulty with all voluntary and passive movement in the legs, such as ankle rotational anomalies, identified as the most frequent cause of lower limb torsional deviations followed by pelvic malalignment (Simon et al., 2015). The consequence of this impairment is shorter strides and difficulties performing rapid changes of direction, which explains the lower scores in both CODA tests.

Class FT6 players showed also significant differences with regard to FT8 players in MAT but not IAT, but both comparisons with large effect sizes (*d* = 1.01−1.23). We should consider the eligible impairments for class FT6: ataxia (impaired control of voluntary movement), athetosis (involuntary contractions of the muscles), or dystonia (sustained muscle contractions that cause twisting and repetitive movements or abnormal postures). FT6 players may have good dynamic balance compared with static balance (IFCPF, 2015b). Athletes with dystonia, athetosis and ataxia, in particular, usually have problems with balance and starting, stopping and turning when running. They also have varying degrees of difficulty with balance while hopping and jumping; with many postural body adjustments for static/dynamic balance. If we compare the CV within each class in the two CODA tests, we observe that MAT test has higher scores. Particularly, FT6 class exhibits a CV of 6.85% in IAT and 17.09% in MAT (10.24% difference between tests), higher than the other FPCP classes (FT5 = 6.45%; FT7 = 1.94%; FT8 = 3%). The main activity limitation in these players is the coordination and movement control, and MAT requires displacements in several directions (frontwards, backwards, and lateral). Their CV is lower than other classes as FT5 or FT7 in IAT, because this athlete usually demonstrates better running mechanics or running pace (IFCPF, 2015b). However, because three different impairments are eligible for this class, large variability is also expected among players within this functional profile.

With regard to class FT7, we should consider again the functional profile of this class: spastic hemiplegia. These athletes usually have activity limitations in gait/running both in stance and swing phase on the impaired side. Foot placement is affected by either weakness in dorsiflexion muscles and/or over-activity in plantar flexor muscles. Knee and hip control are also affected by spasticity and possible loss of range of motion due to contracture. The kinematics of walking in youth and adults with CP (e.g., increased limb asymmetry, reduced stride length, and increased stride time) has been linked to their reduced walking economy (Unnithan et al., 1996). Although the athlete usually walks with a noticeable limp, he may appear to have a smoother stride when running but may not have a consistent heel strike. Asymmetry in the angle of touch down during landing probably resides within a compensation for unilateral neurological impairment of the individual (Kloyiam et al., 2011).

Larger angle of touch down (increased plantar flexion) may result from the fact that the affected limb is less able to conserve energy and stabilize the joints during movement. In MAT test, lateral displacements (toward right and left sides) were required, and these athletes exhibited difficulties in pivoting and balancing on the impaired side. In comparison, no differences between FT7 and FT8 classes in IAT could be explained because displacement is forwards, and a higher degree of plantar flexion allows greater energy conservation in the tendon, and the plantar flexed foot acts like a vertical (Fonseca et al., 2004) contributing to greater joint stability. IPC Athletics (2015) classification rulebook states that in T37 athletes, with similar profile to FT7 in CP Football, a limp may disappear almost totally while they run. The reason is that when walking the leg support during stance phase begins with a heel strike, and this is the most difficult action for athletes with a spastic paresis. When running only the forefoot hits the ground, providing support and push off, and a tight calf muscle facilitates the push off. Therefore, MAT appears here as a better CODA test for the cut-point FT7 vs. FT8 than IAT.

## Conclusions

The IPC is continually developing and refining evidence-based classifications for all sports. No research has systematically investigated the relationship between the CP Football players' functional profiles and their ability to perform CODA tests. The present study makes the following suggestions:

- IAT and MAT could be useful, reliable and valid tests to analyse CODA performance in FPCP, because activity limitation in test performance has been demonstrated compared with controls.
- Comparing between current FPCP classes (IFCPF, 2015b), each test (IAT and MAT) could be applied considering the cut point that classifiers need to make a decision about the FT8 class and other FT classes (FT5, FT6, and FT7).

According to Tweedy et al. (2014) future research should focus on the assessment of the relative strength of association between valid measures of impairment and measures of performance. It is vital that athletes who have positively influenced their impairment scores through effective training are not competitively disadvantaged by being placed in a class for athletes with less severe impairments (Tweedy and Vanlandewijck, 2011). Since CPISRA classification system is based on a functional approach, more research is necessary to develop a sport-specific and evidence-classification system.

Our results can improve the current classification process in CP Football under the new International Federation (IFCPF), and contribute to the description of the current classification profiles, which will be modified and redefined after Rio 2016 Paralympic Games.

## Author contributions

RR: design of the work, data acquisition, analysis, interpretation of data, drafting the work, revising, final approval of the version to be published. JS: design of the work, analysis, interpretation of data, drafting the work, revising. JY: analysis, interpretation of data, drafting the work, revising, final approval of the version to be published. MG: data acquisition, interpretation of data, drafting the work. MC: data acquisition, interpretation of data, drafting the work.

### Conflict of interest statement

The authors declare that the research was conducted in the absence of any commercial or financial relationships that could be construed as a potential conflict of interest.
